# Distance mis-estimations can be reduced with specific shadow locations

**DOI:** 10.1038/s41598-024-58786-1

**Published:** 2024-04-26

**Authors:** Rebecca L. Hornsey, Paul B. Hibbard

**Affiliations:** Department of Psychology, University of Essex, Colchester, UK

**Keywords:** Distance perception, Virtual reality, Attention, Visual cues, Shadows, Human behaviour, Psychology

## Abstract

Shadows in physical space are copious, yet the impact of specific shadow placement and their abundance is yet to be determined in virtual environments. This experiment aimed to identify whether a target’s shadow was used as a distance indicator in the presence of binocular distance cues. Six lighting conditions were created and presented in virtual reality for participants to perform a perceptual matching task. The task was repeated in a cluttered and sparse environment, where the number of cast shadows (and their placement) varied. Performance in this task was measured by the directional bias of distance estimates and variability of responses. No significant difference was found between the sparse and cluttered environments, however due to the large amount of variance, one explanation is that some participants utilised the clutter objects as anchors to aid them, while others found them distracting. Under-setting of distances was found in all conditions and environments, as predicted. Having an ambient light source produced the most variable and inaccurate estimates of distance, whereas lighting positioned above the target reduced the mis-estimation of distances perceived.

## Introduction

Many visual cues to distance are available in natural environments^1^, and the precision and accuracy with which judgements can be made will depend on the particular combination of information available in a given context^2^. For example, Surdick et al.^3^ found that in a stereoscopic display, perspective cues were more effective at producing an accurate perception of distance than other depth cues, while the effectiveness of some lighting cues, such as relative brightness, contributed very little. The effectiveness of many cues, especially parallax distance cues, also tends to reduce rapidly with distance^1,2^.

Perception of distance in sparse environments tends to be poor, as predicted from geometrical considerations of the limited visual cues that are available. For example, distance tends to be underestimated, with relatively high uncertainty, when few visual cues are available^4–9^. We have previously tested whether adding specific environmental cues (binocular cues, linear perspective, surface texture, and scene clutter) could enhance the accuracy and precision of distance-dependent perceptual tasks using virtual reality^10^. Performance in this instance was measured via the degree to which individual cues contributed to a reduction in the amount of bias and variability in participant responses. It was found that adding visual information did indeed improve performance, such that a full-cue context allowed for the highest levels of accuracy and precision. In this case, both binocular and pictorial cues were important for obtaining accurate judgements. To further to these findings, the current experiment assessed the specific contributions made by clutter and cast shadows to the perception of relative distance, to explore the importance of lighting cues in complex environments for the accurate perception of 3D space.

Shadows occur when an object or surface disrupts the visibility of another surface or object to a light source, thus reducing the degree to which it is illuminated. The surface in shadow may belong to an object that is the same as, or different from, the one that is responsible for the shadowing. Mamassian et al.^11^ provide definitions for a number of distinct aspects of shadows and shading, including shading, attached shadows, and cast shadows. *Shading* refers to the variation in the amount of light reflected by surfaces, as a result of their orientation relative to the light source. Shading may be distinguished from the shadowing effect under consideration in the presented experiment, which is a result of the occluding of regions of surfaces from the light source. *Attached shadows* are those that are formed on the same surface that is occluding the light source. In contrast, *cast shadows* are those which are formed on a different surface from the one occluding the light source. It is cast shadows that are the focus of the current experiment, and in particular shadows that are cast on a horizontal surface (such as a ground plane or table top) by objects that are not attached to the surface.

**Figure 1:**
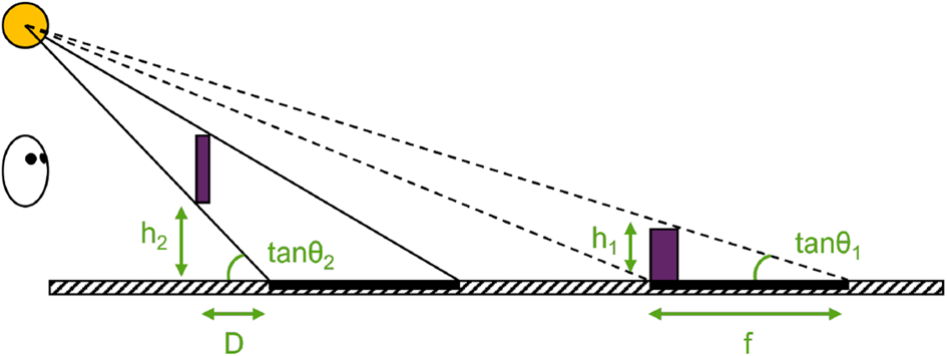
Examples of how the light source location can be more easily determined when there are multiple shadows from additional objects within the scene. Dashed lines identifying the light occluded by objects on the ground surface, where shadows and the objects are attached; solid line identifying the light occluded by the floating object, where the cast shadow is not attached. The distance of the light source to each object, and their relative heights, has an impact on the length of their cast shadows. Representation of Eqs. (1) and (2).

When an object is placed on a surface, its cast shadow will be directly adjacent to the object in the image, as shown in Fig. 1. In this case, the size of the shadow, relative to the height of the object, can be used to provide information about the direction of the light source. For an object with a height (h_1_), and a shadow with a length (f), the direction ($\theta _1$) of the light source is given by:

1 $$\begin{aligned} \mathtt {tan} \theta _1 = \frac{\mathtt{h}_1}{\mathtt{f}} \end{aligned}$$

**Figure 2:**
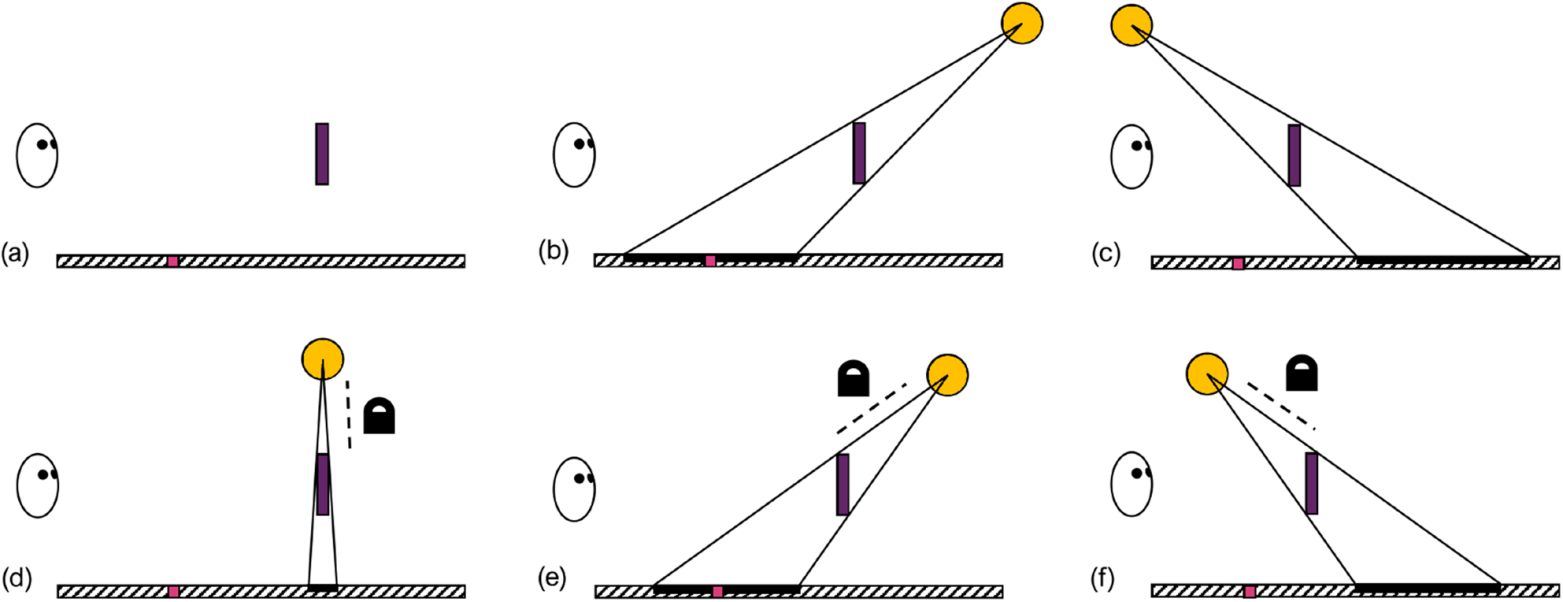
Examples of the shadow conditions where the light source is locked to the target (**a**) Ambient; (**b**) Static back; (**c**) Static in front; (**d**) Locked on top; (**e**) Locked back; (**f**) Locked in front. The light location moves in sync with the adjustments made to the target object location. Light source in three locations compared to observer, purple target object, pink reference line, black shadow on surface.

When an observer views an object above a table-top at eye-height, and the object is illuminated from a single direction, the shadow will tend to be detached from the object, examples of this are presented in Figs. 1 and 2. When the light source is directly above the object, its shadow will be cast on the horizontal surface at the same distance as the object. If the light source if further away than that the object, the shadow will be cast at a distance that is nearer than the object itself. Conversely, if the light source is nearer than the object, or behind the observer, the shadow will be cast at a distance that is further away than the object. The location of the shadow on the surface provides information about the distance of the object relative to locations on the surface, albeit in a way that is ambiguous due to its dependence on the direction of the light source. Geometrically, it can be seen that, when the shadow is further away than the object, its distance from the object in the horizontal direction is given by:

2 $$\begin{aligned} \mathtt {D}=\frac{\mathtt{h}_2}{\mathtt {tan} \theta _2} \end{aligned}$$

where D is the distance, h_2_ is the height of the object and $\theta _2$ is the direction of the light source. Thus, if the direction of the light source can be estimated, the distance of the object along the surface, relative to the location of its shadow, can be inferred.

Allen^12^ demonstrated that shadows can be used a source of information about the distance to objects, by disambiguating the contributions of distance and vertical location to the height in the visual field of objects. In addition, another study by Allen^13^ showed that the direction of cast shadows contributes to distance judgements, and that shadows have also been found to improve distance judgements regardless of the direction of lighting^14^. Cavanagh et al.^15^ showed that the lateral displacement of a shadow from a target object influenced the perceived depth between the two; therefore, for a given depth separation, the size and direction of this offset will be determined by the direction of lighting. The results obtained were consistent with both a model in which the visual system assumes a single light source^16^, and a Bayesian model that weighted different lighting directions by their relative likelihood in the natural environment.

Wanger et al.^17^ used two tasks to assess the contribution of shadows to the perception of 3D surface layout. They found that shadows improved observers’ ability to accurately align objects in three-dimensional space, and to accurately match their size. Additionally, te Pas et al.^18^ conducted an odd-one-out experiment, in which participants were presented with three stimuli with varying lighting and shadows. By collecting data of participant’s eye fixations, reaction time, and percentage of correct answers they showed that participants relied primarily on shadows in identifying differences in the direction and intensity of lighting.

Cast shadows can also have a strong influence on the apparent 3D layout and motion in moving scenes^19–22^. Kersten et al.^19^ showed that when presented with a ball moving linearly away from the observer in depth, and a shadow which would correspond with a bouncing ball, an observer was more likely to use the shadow information to perceive the ball as having non-linear movement. One of the conclusions of this study was that when a shadow moves, observers assume that this is caused by the movement of the objects, rather than the light source. That is, the locations of shadows are used to inform the estimation of the locations of objects under the assumption of a fixed light source.

Dee et al.^23^ reviewed the literature on the visual effects of shadows. For simple cases with an object on a flat surface, when a light source does not move, then physical properties of the object such as its size, motion, and shape can be inferred from the shadow.

Shadows have great potential for improving the perception of distance in augmented reality, by helping users to accurately locate virtual objects within the physical environment. In virtual reality, Hu et al.^24^ showed that cast shadows can be used to determine when an object is in contact with a tabletop, or to judge the distance between the two. The presence of cast shadows can increase the accuracy of distance judgement in augmented reality^25,26^, and this is affected by the degree of mismatch in the direction of lighting in the real and virtual environments^25,27^.

The current study had three goals. The first was to determine whether the presence of cast shadows could improve the accuracy of distance judgements for an object that was located above a horizontal surface. The second was to determine how these distance judgements were affected by the position of the light source, which governs the spatial location of the shadow relative to the target object. The third goal was to determine whether accuracy could be further improved by the presence of visual clutter on the tabletop, which could be used to determine the direction of the light source, and thus allow the shadow to serve as an unambiguous cue to distance.

The experiment consisted of two virtual environments which were tested separately, both using a perceptual matching task in a VR headset. Participants were tasked with matching the distance of a floating target object to that of a horizontal reference line on a tabletop. The location of the light source was varied between blocks.

In one lighting condition, only ambient lighting was present, so that no shadows were cast. In all other conditions, one light source was present, and its location relative to the object was varied. This light source was either in a fixed location in the environment so that it did not move with the object, or it was locked to the object, so that it moved and maintained a constant position relative to the object. In the fixed light condition, the light source was either at the near (observer’s) end of the table or the far end. For the conditions in which it was locked to the object, the light was either nearer, directly above, or further away than the object. These lighting conditions are depicted in Fig. 2. The experiment was repeated in a Sparse and a Cluttered environment.

The first hypothesis relates to the overall performance in the Sparse and Cluttered environments: (1) regardless of the influence of shadows, the presence of additional visual cues in the Cluttered environment compared to the Sparse environment is predicted to improve the accuracy of distance judgements^10,28^.

In the Sparse environment, shadows are predicted to have a number of effects. As shadows are expected to contribute to distance estimation, the first hypothesis here is that (2) performance will be worse overall in the Ambient condition. In addition, (3) performance is expected to be more precise for the condition in which the light source is directly above the target (Locked on top), when it is perfectly aligned with the reference at the correct distance, than when the light source is in front or behind. This is because in this condition, the observer is required to align two features (the shadow and reference line) that overlap, rather than alignment with a spatial offset. Furthermore, (4) performance is hypothesised to be more accurate when the light source is locked to the moving object than when it is static in the environment, since this creates a constant shape and offset of the shadow. While the shadow provides a potentially useful cue to the location of the object, when the light is not directly above the object there will be an offset between the reference line and the shadow when the object and reference are perfectly aligned. Additionally, it is hypothesised (5) that observers may have a tendency to align the shadow with the reference line. This would lead to an the object being positioned nearer when the light is in front of the object than when it is behind it.

Finally, (6) it is hypothesised that conditions which improve the accuracy of settings will be more evident in the Cluttered environment than in the Sparse, since this provides more reliable information about the direction of the light source, from the sizes of the shadows cast by the surrounding objects on the table-top (Eq. 1). This should also reduce any systematic biases that are associated with the lighting direction. Note that this predicted increase in the effects of shadows is in addition to the overall improvement in performance in the cluttered environment relative the sparse environment.

**Figure 3:**
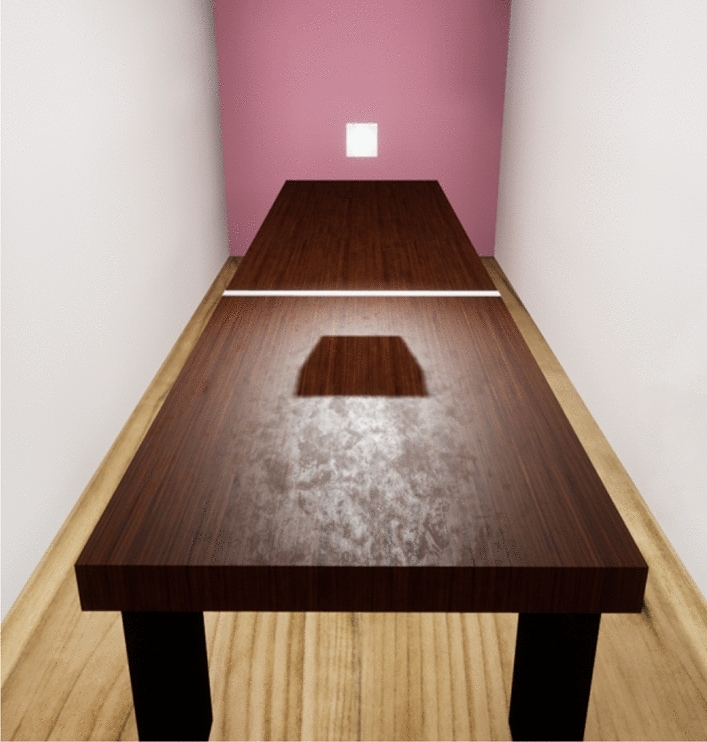
View from above: the Sparse environment, where the target object is floating above the tabletop, the reference line is visible on the tabletop, and the cast shadow is also visible.

## Methods

### Participants

Total of 21 naive participants were recruited using the University’s online system as well as through word of mouth. Participants received a £5 voucher for approximately 45 minutes of their time to complete the study.

The methods and protocols were approved by the Psychology Ethics Officer and carried out in accordance with the ethical guidelines at the University of Essex. All participants gave informed consent.

### Materials and apparatus

**Figure 4:**
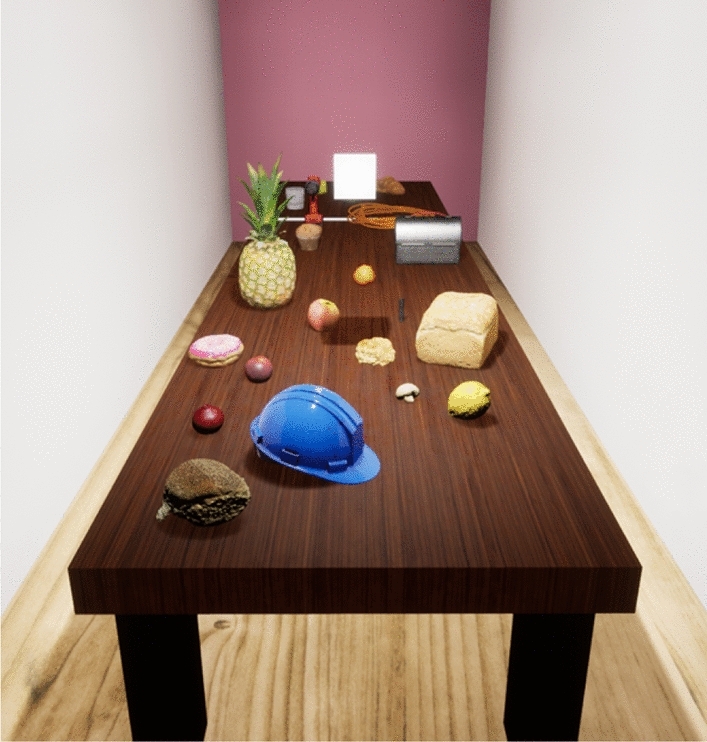
View from above: the Cluttered virtual environment, where additional objects are positioned randomly on the tabletop, casting their own shadows.

An Oculus Rift headset and associated controllers were used, with two environments created in Unreal Engine 4.2. Figure 3 shows the virtual Sparse environment presented to participants: a room containing just a long table (1 m width by 4 m length) and the target object (an 10 by 10 by 5 cm cuboid presented at eye-height). The architecture of the room, target, and reference line were created using the Engine’s Starter Content. The Cluttered environment incorporated objects added on to the tabletop, shown in Fig. 4. These were scans obtained from Unreal Engine Marketplace^29,30^ or real objects scanned by the experimenters. A total of 21 objects were positioned randomly across the full length of the table surface.

The target object had a point light locked to the participant-facing side, with an intensity of one lumen and attenuation radius of 10 cm. This light was programmed to not cast any shadows during the experiment, and ensured that the luminance of the target object itself did not differ across the lighting conditions of the experiment, which did however affect the presence, location and shape of the cast shadows.

### Shadow conditions

Both experiments contained the following six shadow-lighting conditions, depicted in Fig. 2:

- Ambient: no point light source, no cast shadows.
- Static back: a light source locked to the edge of the table furthest from the participant (position in environment: X = 36.7, Y = 0, Z = 175 cm; rotation: X = 360 °, Y = $-65$ °, Z = 180 °). The size and shape of the shadow changed as a function of the distance between the target and the light source, which was positioned between the target and the far edge of the table.
- Static in front: a stationary light source, positioned at the edge of the table closest to the participant (position in environment: X = 37, Y = 0, Z = 175 cm; rotation: X = 0 °, Y = $-65$ °, Z = 0 °). The size and shape of the shadow varied as a function of the distance between the target and light source, which was positioned between the target and participant.
- Locked on top: a light source attached to the target object (position from target: X = 0, Y = 0, Z = 120; rotation pointing directly down onto target). The cast shadow was directly below the target.
- Locked back: a light source attached to the target object (relative transformation position from Locked on top: X = $-60$, Y = 0, Z = 0 cm; rotation: X = 0 °, Y = 50 °, Z = 0 degrees °). The cast shadow moved with the target object and was positioned between the target and the far edge of the table.
- Locked in front: a light source attached to the target object (relative transformation position from Locked on top: X = 60, Y = 0, Z = 0 cm; rotation: X = 0 °, Y = $-50$ °, Z = 0 °). The cast shadow moved with the target object and was positioned between the participant and target

In Fig. 2b,c the light is static in the environment, whereas in (d), (e), and (f) the light source is attached to the target object and moves along with the distance adjustments to the target. There was no point light present in (a). The light sources were obtained from Unreal Engine’s Starter Content.

The conditions were presented in separate blocks, each having 20 trials. In the Cluttered environment, the objects on the virtual table cast their own shadows from the light sources; these were not manipulated in any way. The light sources were not visible objects themselves, rather the emitted light was visible only on the objects within the environment.

### Task and procedure

The goal was for each participant to match the distance of the target object (a floating box) to that of a reference line on the table surface. Participants were told to read instructions and these were also described to them by the experimenter; there was an opportunity to ask questions before starting. The order of the environments was counterbalanced, alternate participants were allocated to start in either the Cluttered or Sparse environment. In all conditions the task was to move the target object so that it aligned in distance with the reference line on table.

Once inside the VR headset participants adjusted the height of a chair to ensure targets appeared at eye-height, and sat in an indicated area for the duration of the experiment. They were instructed to not move from this position during the experiment. A horizontal reference line appeared on the surface of the table in front of the observer at a random distance between 40 and 365 cm. The target object was positioned at eye-height and, using the controller’s A and B face-buttons, participants could move the target along the X axis (forwards/backwards in egocentric distance) to a position where it looked as though it was directly above the reference line. Once the distance was decided upon, the participant pressed the controller’s trigger which saved the trial and moved onto the next one. The next trial automatically began by randomising the distance of the reference line and target box. No indication was given to participants about when the condition was to change, and no feedback on the accuracy of their settings was provided.

The lighting condition was altered across blocks of trials. The metrics recorded were the final position participants set the target object to be at in each trial and the position of the reference line, so that the offset for each trial could be calculated.

## Results

### Formatting data

The raw data output from the experiments were in a comma separated value format; each session had an independent .csv file named by the experiment name, time, and date of the session. Each row represented an individual trial with columns for the condition number, the distance of the reference line (in cm), and the set distance of the target (in cm). A numerical key was used to identify the conditions.

The signed and unsigned error for each trial was calculated by subtracting the distance of the reference stimulus from the set distance of the target stimulus. For the signed errors, a positive value indicated that the object was positioned further away than the references, and a negative value indicated that it was positioned closer than the reference. Unsigned errors reflect the overall error in settings, incorporating both systematic biases and random variability. Signed errors reflect any systematic biases in the settings, since random variability will tend to cancel out across repeated trials.

The following exclusion criteria were decided upon before the experiment: any participants who did not finish the entire experiment, and any participant who withdrew their data after a two-week window from completion. Additionally, removal of trials where the set distances were further or closer than the ends of the table were to be excluded, but no data were set beyond these limits (see Section “Evaluation of the methodology”). No data were excluded based on these criteria.

### Analysis strategy

A Pearson’s correlation was conducted, separately for the two environments, on the distance of the reference line and set target positions in each trial to identify the general relationship between participant estimates and correct settings.

A linear mixed effects model was used to analyse the data with the following equation, where the distance set, by participants (P) in each trial, is predicted by the distance of the reference line (D) and the lighting condition (L, numbered from 1 to 6):

3 $$\begin{aligned} \mathtt {Distance Setting} \sim \mathtt {D} + \mathtt {L} + \mathtt {D} * \mathtt {L} + ( 1 + \mathtt {D} \mid \mathtt {P}) \end{aligned}$$

Two-way (environment-by-condition) repeated measures ANOVAs were used to identify how these factors influenced the unsigned and signed distance-setting errors. These were followed up with one-way repeated measures ANOVAs to assess how settings were affected by the shadow condition in each environment, Tukey’s HSD to which pairs of conditions differed.

### Analyses

**Figure 5:**
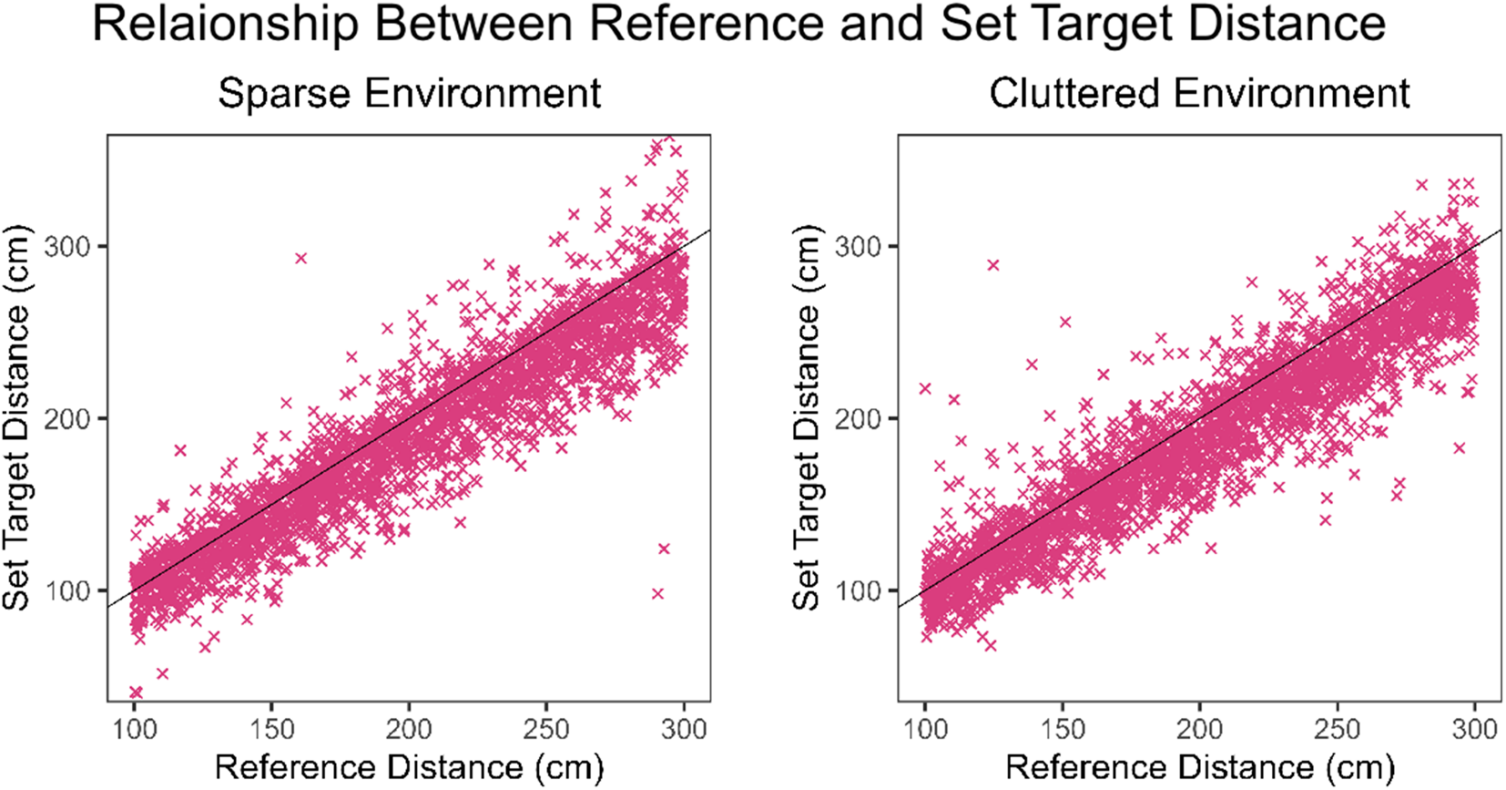
Scatter plot of the relationship between the distance of the reference line and the set target distance in each trial. Black line represents accurate performance with an intercept of zero and a slope of one.

**Figure 6:**
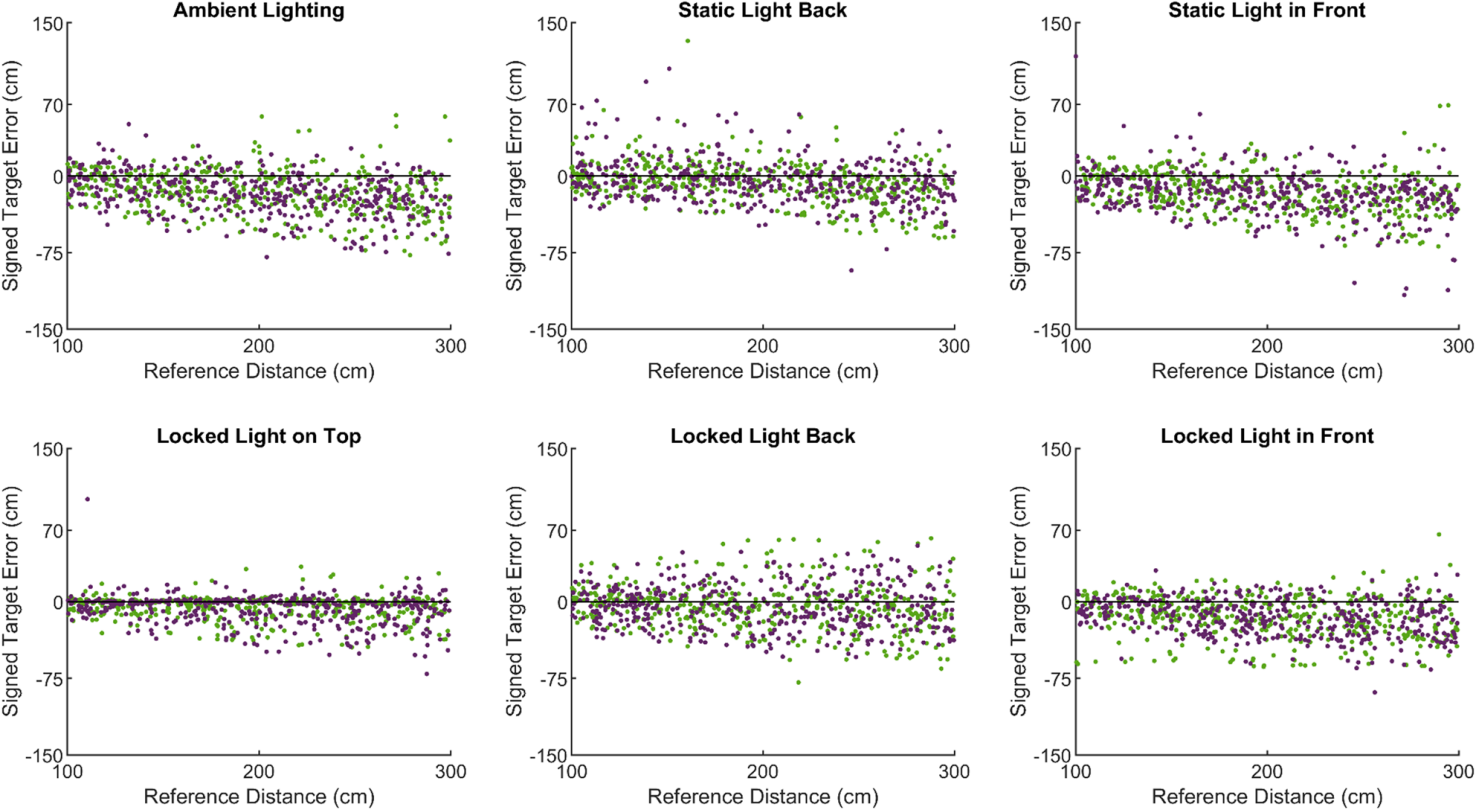
Scatter plot of the mean signed errors in the position of the set target in each condition and environment. Errors from the Sparse environment in green and Cluttered environment in purple.

There was a consistent under-setting of distances, in that regardless of the lighting or sparsity of the context, participants set the target to be not as far away as it should have been, which can be seen in Figs. 5 and 6. A correlation was conducted on the distance of the reference line and set target positions, to determine the overall relationship between the estimates and correct settings. In the Sparse environment, there was a strong positive relationship between the two (r = 0.935, *p*<0.001); the same positive trend was found in the Cluttered environment (r = 0.933, *p*<0.001).

The data for each environment were analysed separately: Table 1 presents the slope and intercept information for the lighting conditions from both environments. Accurate performance would produce a slope value of one and an intercept of zero. In both environments the intercept was largest in the Static back lighting condition, and closest to zero in the Locked in front condition. All slopes were below one, indicating under-setting of distances. The closest to accurate slope estimate was produced by the Locked on top condition in the Sparse environment, and Locked back in the Cluttered environment.

#### Unsigned error

**Figure 7:**
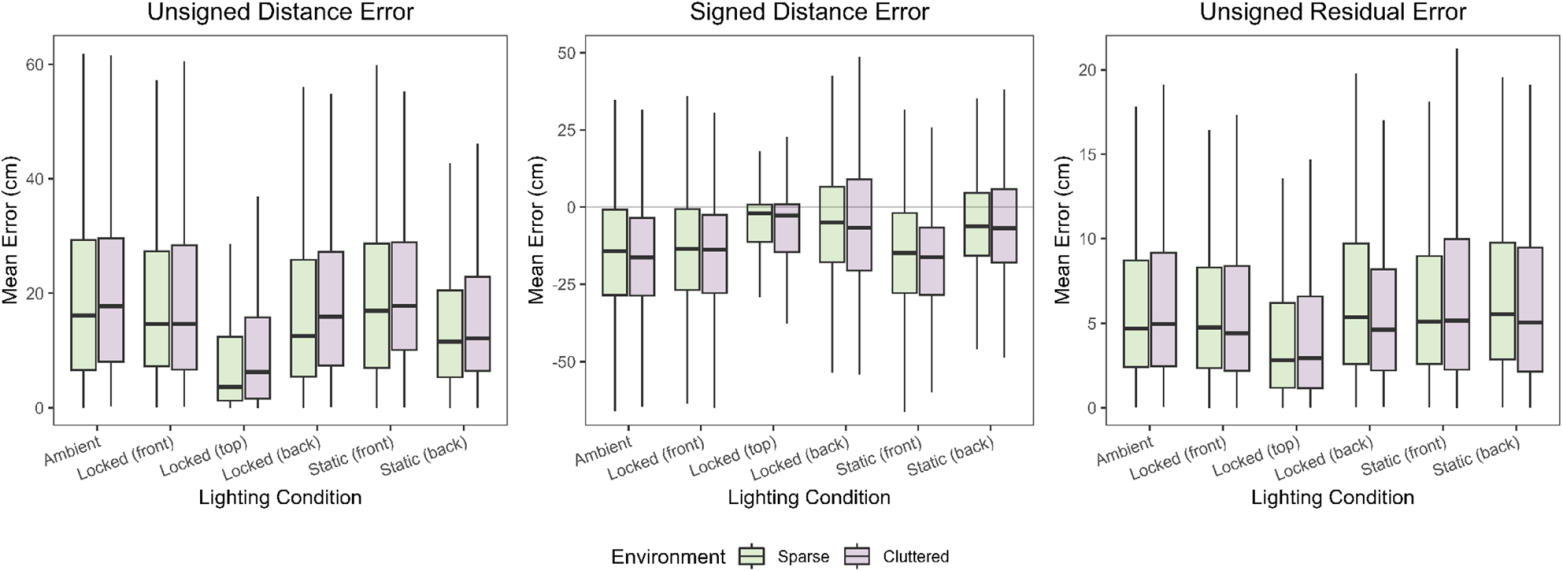
Box and whisker plots showing the distribution of errors from the six lighting conditions. The average unsigned and signed error for the Sparse environment in green; Cluttered environment in purple.

Unsigned errors, which reflect the overall accuracy of the settings, are plotted as a function of environment and condition in Fig. 7. A two-way repeated measures ANOVA showed a significant effect of condition (F (5, 100) = 12.187, *p*< 0.001) but no effect of environment nor interaction . Overall performance, and the effects of shadows, were thus no more different in the Cluttered environment (hypotheses 1 and 6).

The effects of shadows were analysed separately for the sparse and cluttered environments. Unsigned errors were significantly influenced by the lighting conditions in both the sparse (F (5, 100) = 9.11, *p*< 0.001) and cluttered (F (5, 100) = 7.47, *p*< 0.001). The only conditions with consistently smaller unsigned errors was the lit from above condition, which were significantly smaller than in all other conditions (all *p*< 0.02).

These results show that performance was most accurate when the light was directly above the target, and the shadow directly aligned with the reference when the object is at the correct location (hypothesis 3). However, there was no evidence that unsigned errors were reduced in any of the other shadow conditions (hypothesis 2).

#### Signed errors

Signed errors, which reflect systematic biases in the settings, are plotted as a function of environment and condition in Fig. 7. A two-way repeated measures ANOVA showed a significant effect of condition (F (5, 100) = 19.815, *p*< 0.001) but no effect of environment nor interaction . Overall bias, and the effects of shadows, were thus no different in the Cluttered environment (hypotheses 1 and 6).

The effects of shadows were analysed separately for the two environments. Signed errors were significantly influenced by the lighting conditions in both the Sparse (F (5, 100) = 10.6, *p*< 0.001) and Cluttered (F (5, 100) = 12.7, *p*< 0.001).

It was predicted that, if observers had a tendency to align the shadow with the reference, then settings should be closer when objects were lit from in front than behind (hypothesis 5). This was found for both environments, and for both the static lights and those that were locked to the target objects (all *p*< 0.05).

#### Residual errors

Using the data from Table 1, the residual errors for each participant in each environment and condition were calculated. These were calculated by performing a regression of set distance on reference distance for each participant and each condition, then calculating the average absolute difference between each setting and the values predicted by the regression. This separates out the between-trial variation in settings from the systematic error captured by the regression. The mean unsigned residuals were then used in two way repeated measures ANOVA. A significant effect of condition was found (F (5, 100) = 3.654, *p* = 0.004), but no effect for environment nor interaction. The distribution of these data are shown in Fig. 7. Residual errors were lowest when the light source was locked to the top of the object.

**Table 1:** Regression Analyses of the distance perceived in each lighting condition in the two environments showing estimates, standard errors (SE) and 95% confidence limits (CLs), using Eq. (3). Accurate performance would have an intercept of zero and a slope of one. The *p* value indicates whether the row is significantly different from the values of the Ambient condition.

Condition		Intercept estimate	SE	*T*	Intercept CLs	*p*
Ambient	Sparse	6.199	3.698	1.676	[− 1.052 13.449]	
	Cluttered	5.822	4.112	1.416	[− 2.241 13.855]	
Locked in front	Sparse	− 0.485	3.162	− 2.114	[− 6.685 5.715]	0.035
	Cluttered	− 0.986	3.387	− 2.010	[− 7.629 5.656]	0.045
Locked on top	Sparse	2.987	3.115	− 1.031	[− 3.121 9.095]	0.303
	Cluttered	3.865	3.364	− 0.586	[− 2.732 10.452]	0.561
Locked back	Sparse	10.129	3.132	− 1.031	[3.988 16.270]	0.210
	Cluttered	3.120	3.298	− 0.819	[− 3.347 9.587]	0.413
Static in front	Sparse	7.238	3.123	0.333	[1.114 13.362]	0.739
	Cluttered	5.536	3.308	− 0.086	[− 0.950 12.022]	0.931
Static back	Sparse	16.005	3.106	3.157	[9.914 22.096]	0.002
	Cluttered	9.749	3.271	1.201	[3.3349 16.163]	0.230

		Slope Estimate	SE	T	Slope CLs	*p*
Ambient	Sparse	0.891	0.020	44.311	[0.852 0.931]	
	Cluttered	0.885	0.017	52.008	[0.852 0.919]	
Locked in front	Sparse	0.925	0.0152	2.250	[0.895 0.955]	0.025
	Cluttered	0.930	0.016	2.783	[0.898 0.961]	0.007
Locked on top	Sparse	0.952	0.015	4.048	[0.923 0.982]	<0.001
	Cluttered	0.943	0.016	3.551	[0.911 0.974]	<0.001
Locked back	Sparse	0.922	0.015	2.044	[0.892 0.951]	0.001
	Cluttered	0.959	0.016	4.616	[0.928 0.990]	<0.001
Static in front	Sparse	0.887	0.015	− 0.266	[0.857 0.917]	0.026
	Cluttered	0.885	0.016	− 0.045	[0.854 0.916]	0.964
Static back	Sparse	0.888	0.015	− 0.222	[0.858 0.917]	0.824
	Cluttered	0.928	0.016	2.704	[0.897 0.959]	0.007

## Discussion

This experiment aimed to identify which cast-shadow conditions produced the most accurate responses in a perceptual matching task, and whether the addition of scene anchors enhanced the influence of cast shadows. Six lighting conditions were created and presented in virtual reality. The task was repeated in Cluttered and Sparse environments. A thin box was used as a target so that the most visible face was flat and therefore did not have shading as an added visual cue. The presence of scene clutter did not improve the accuracy of settings, and did not affect the influence of shadows on these settings. A light source directly above the target, projecting a shadow at the same distance as the object onto the table-top, produced the most accurate settings. When the light source was in front of or behind the target, there was a tendency for observers to align the shadow with the reference line.

### Interpreting the findings

There was a strong correlation between the reference stimulus location and set target location, showing that participants understood the task and completed it appropriately. There was an overall tendency however, for participants to set the target stimulus at a shorter distance than the reference line Fig. 7. This indicates an over-estimation of target distance relative to the reference line, which may reflect a partial misinterpretation of its greater height-in-the-field as a cue to distance^1,2^. This effect has been found for similar contexts in augmented reality^31^.

No differences in accuracy or precision were identified between the environments. However, a difference was found between the lighting conditions. The overall error in responses for each condition can be seen in Fig. 6, where the Locked on top lighting clearly produces the most precise distance estimates. This is supported by the unsigned errors made, Fig. 7, which were lowest in this condition.

### Evaluation of the methodology

Initially, an exclusion criterion was to remove any trials with set distances outside the range of the table length, as this would not be possible should the experiment have been completed correctly. However this exclusion criterion was not used, because in the hypothesised event that the shadow was visible and being aligned with the reference at either end on the table, but the target was subsequently located beyond, this would have led to the removal of trials in which the participant was following a coherent strategy.

Lighting conditions experienced in daily life tend to include ambient light from overcast weather diffusing the light source, and directional lighting, for example from the sun or from ceiling lights^32^. The lighting conditions used here were created to replicate these, with overtly visible cast shadow effects (with the notable exception of the Ambient lighting condition). Additionally, some less common light instances were created, although all were physically plausible.

We hypothesised that performance would be more most accurate when the position of the light source was locked to the target objects, so as to produce a constant offset in the location of the shadow. While light sources that are a fixed location produce more complex shadow effects, these are also more likely to be encountered in everyday life, and that participants may be more familiar with this dynamic shadow cues. We might also predict that the additional shadows present in the cluttered condition might be most useful when the light source was moving with the target object, by providing information about the movement of the light source.

Retinal size is a reliable cue when either the dimensions of a stimulus or the distance is known by the observer^33,34^. Here, the participants were not familiarised with either the dimensions of the target, or the testing area within the virtual environment. The participants were not primed as to whether the size of the target would be consistent or changing throughout the experiment. However, if they assumed the target was of a constant size, then its retinal size provides a reliable cue to changes in distance, which are likely to contribute to the reliable distance estimation shown in Fig. 5.

The reference stimulus was presented at a randomly chosen distance on each trial, unlike many other distance-matching studies where the target appears at a limited few distances^35–37^. This avoided the possibility of participants remembering the relative size of the target at each set distance, and then try to replicate the size, rather than complete the trial as required^38^.

The two environments were created so that a comparison of whether scene clutter aids in the perception of distances. The specific objects used in the Cluttered environment were chosen as they are typical everyday objects that varied in size and shape so that the shadows produced would also be varied. The positioning for these objects was random, a structured approach to the positioning of the clutter may influence the perception of the scene.

Clutter objects and an abundance of shadows on the tabletop may have been distracting for some participants, which may have contributed to the non-significant difference between the two environments. After discussions with some of the participants it became apparent that a subset of the participants valued the objects in the Cluttered environment differently than other participants. In particular, individuals with attention deficit hyperactivity disorder may find the additional objects in the Cluttered environment too distracting. Thus, increasing the availability of extra visual cues may not always lead to the anticipated improvements in performance^39^.

### Practical implications

Sugano et al.^27^ showed that shadows are an important depth cue in AR, but that lighting needs to be consistent with physical lighting due to the see-through nature of the device. Gao et al.^25^ used an AR device to test the impact of shadows on distance perception. It was predicted that distance would be under-estimated, that virtual shadows would enhance accuracy in the perceptual matching task, and that misalignment between physical and virtual lights would however impair these estimates. Our results also showed a misestimation of distances and that lighting conditions also had a significant impact on these judgements. Our results are thus consistent with those of Gao et al.^25^, in that we found an improvement in performance, shown in the reduction in signed, unsigned and residual errors when objects were lit from above. This is consistent with other studies that have that the presence of these ‘drop’ shadows support the most accurate perception of distance in AR^40^. In our VR study we did not consider the possibility of an inconsistency in the shadows created for different objects. This is an important practical implication for AR, in which some shadows will be created by natural light sources in the real world, and others by virtual light sources. This misalignment can negatively affect the accuracy of distance judgements^25^.

The lit from above condition in this experiment, where the light was attached to and moving with the object, is a special case in that it creates a situation in which the shadow is cast on the horizontal plane at the same distance as the object. Moreover, the fact that it moves with the object may mark it out as an additional light source, thus potentially reducing any effects of conflict between this and other light sources in the virtual scene. As such, this condition is likely to also improve accuracy in augmented reality applications.

### Conclusions

Adding clutter to an environment, where there are more shadows cast and more points of reference, does not enhance the accuracy of distance estimates within the scene compared to an identical environment minus the clutter. Clutter objects are therefore not used as points of reference, or at least there are some subsets of participants who do not find them useful for improving performance.

Consistent with previous literature, there was an under-setting of distances throughout the experiment. From the lighting techniques tested, both the precision and accuracy were highest in the Locked on top lighting condition. This condition provides the observer with a simple strategy of aligning the shadow with the reference line in order to complete the task accurately. Differences in biases between the cases where the light was in front of or behind the object suggest that observers did in fact have tendency towards aligning the shadow and reference line, even in cases where lighting was not from directly above.

Some of the lighting conditions presented in this experiment can be considered to be ‘unnatural’ in typical viewing of physical space, as there are few instances where a light source is attached to an object with some sort of offset. The two static lighting conditions are more representative of typical viewing. However, implementations of unnatural lighting can be seen in specific applications of VR and cinematography. It is in these instances where the perception of distance can be enhanced through the use of the conditions presented in this experiment.

## Data Availability

The datasets generated and analysed during the current study are available in the University of Essex Research Data Repository, http://researchdata.essex.ac.uk/143/
